# Sepsis-Induced Exosomal Transfer of *MAFB* mRNA Reprograms Hepatocytes via a miR-155–*Jarid2*–*H3F3A* Epigenetic Cascade

**DOI:** 10.3390/cells15161481

**Published:** 2026-08-18

**Authors:** Gizaw Mamo Gebeyehu, Milorad Zjalic, Rita Bognár, Benjámin Farkas, Shima Rashidiani, Géza Makkai, Tibor Z. Jánosi, Péter Urbán, József Kun, Attila Gyenesei, Marianna Pap, Željko Debeljak, Marija Heffer, Tibor A. Rauch

**Affiliations:** 1 Institute of Biochemistry and Medical Chemistry, Medical School, University of Pecs, 7624 Pecs, Hungary; 2 Department of Medical Biology and Genetics, Faculty of Medicine, Josip Juraj Strossmayer University of Osijek, 31000 Osijek, Croatia; 3 Institute of Physiology, Medical School, University of Pecs, 7624 Pecs, Hungary; 4 Nano-Bio-Imaging Core Facility, University of Pecs, 7624 Pecs, Hungary; 5 Hungarian Centre for Genomics and Bioinformatics, Szentágothai Research Centre, University of Pecs, 7624 Pecs, Hungary; 6 Department of Pharmacology and Parmacotherapy, Medical School, University of Pecs, 7624 Pecs, Hungary; 7 Department of Theoretical Health Sciences and Nursing, Faculty of Health and Sport Sciences, Széchenyi István University, 9026 Győr, Hungary; 8 Department of Pharmacology, Faculty of Medicine, Josip Juraj Strossmayer University of Osijek, 31000 Osijek, Croatia; 9 Clinical Institute of Laboratory Diagnostics, Osijek University Hospital, 31000 Osijek, Croatia

**Keywords:** exosome, intercellular communication, MAFB, miRNA155, Jarid2, H3F3A

## Abstract

Exosomes carry bioactive macromolecules driving sepsis pathogenesis, but the mechanisms underlying macrophage-to-hepatocyte communication during systemic inflammation remain poorly understood. We investigated how sepsis-induced macrophage exosomes are involved in remote intercellular crosstalk with hepatic cells via transcription factor-encoding mRNA cargo. Human monocytic THP-1 macrophages were stimulated with lipopolysaccharide (LPS), followed by exosome isolation, recipient cell uptake verification, and high-throughput RNA sequencing cargo analysis. To functionally reconstruct downstream signaling in recipient cells, exosome-enriched *MAFB* mRNA was transiently overexpressed in a HepG2 cell model, with subsequent expression changes mapped at both the transcript and protein levels using quantitative PCR and Western blot analyses. This ectopic *MAFB* expression directly upregulates the expression of microRNA-155 (miR-155). Crucially, elevated miR-155 acts as a post-transcriptional repressor that directly targets and downregulates *JARID2* and *H3F3A* mRNAs and their corresponding protein products within the liver cells, orchestrating a “repressor-of-repressors” disinhibition cascade that drives net chromatin remodeling and activation of downstream hepatic target genes. This study demonstrates that sepsis alters exosomal transcription factor mRNA cargo and delineates a mechanistic downstream pathway—MAFB → ↑miR155 → ↓Jarid2 & ↓H3F3A → Chromatin Remodeling → Downstream Hepatic Gene Activation pathway—that provides novel, specific molecular checkpoints for therapeutic intervention in sepsis-induced liver injury.

## 1. Introduction

During an infection, the liver plays a critical role in balancing immunity and metabolism [1]. In the acute hyperinflammatory phase of sepsis, the liver is exposed to high levels of circulating cytokines and pathogen-associated molecular patterns, such as LPS [2]. This inflammatory stress impairs the metabolic and detoxification functions of hepatocytes, while also promoting microvascular thrombosis and focal tissue injury. These alterations are driven by dysregulated communication between activated immune cells and the hepatic microenvironment, highlighting the need to identify the molecular mediators of this distal organ damage. While classical paracrine and endocrine signaling via soluble proteins have been extensively characterized, extracellular vesicles—particularly exosomes—have emerged as crucial mediators of long-range intercellular crosstalk [3]. Exosomes are endosomally derived nano-vesicles (30–150 nm) packed with a selective cargo of proteins, lipids, and functional nucleic acids, including microRNAs (miRNAs) and messenger RNAs (mRNAs) [4]. During severe systemic inflammation and sepsis, macrovascular and immune cells actively shed exosomes into the circulation. These inflammatory exosomes act as vehicles for horizontal gene transfer, protecting fragile RNA species from degradation by extracellular RNases. Upon reaching target organs, such as the liver, exosomes are internalized by recipient hepatic cells via endocytosis or membrane fusion [5]. This delivers their functional macromolecular cargo directly into the cytoplasm, bypassing conventional surface receptor–ligand interactions and initiating rapid phenotypic transitions in the recipient tissue.

The v-maf musculoaponeurotic fibrosarcoma oncogene homolog B (*MAFB*) is a basic leucine zipper (bZIP) transcription factor encoded by a single exon [6]. It belongs to the evolutionarily conserved Maf family of transcription factors, which share a highly conserved DNA-binding domain that recognizes the canonical Maf recognition element consensus sequence [7]. Functionally, MAFB plays a non-redundant role in myeloid lineage commitment, terminal differentiation, and the preservation of an anti-inflammatory phenotype. Within mature macrophages, MAFB serves as a critical checkpoint repressor of pro-inflammatory gene expression, actively suppressing the transcription of inflammatory cytokines while simultaneously driving tissue repair programs and metabolic homeostasis [8].

The profound systemic changes observed during sepsis are driven by rapid alterations in the cellular transcriptional landscape. Beyond classical transcription factors, epigenetic mechanisms serve as critical regulators that dictate how immune and metabolic pathways respond to acute infectious stress [9]. Central to this epigenetic network are miRNAs, which fine-tune gene expression post-transcriptionally without altering the underlying DNA sequence. The orchestrating dynamics of severe systemic inflammation are heavily governed by non-coding RNAs, among which microRNA-155 (miR-155) stands out as a dominant driver of the septic response [10]. As an essential component of the innate immune response, miR-155 acts as a potent amplifier of the ‘cytokine storm’ characteristic of sepsis [11]. Its upregulation directly correlates with increased production of major pro-inflammatory mediators, including tumor necrosis factor-alpha (TNF-alpha) and interleukin-6 (IL-6), effectively preventing the immune system from transitioning back to a homeostatic state [10,12].

In the context of the liver—a primary metabolic and immunological hub during sepsis—elevated levels of miR-155 distort normal homeostatic functions and drive cellular dysfunction. Critically, miR-155 does not operate in isolation; it directly modulates the stability and translation of transcripts encoding key epigenetic modifiers. The interplay between microRNA dynamics and chromatin-remodeling complexes forms a highly cooperative regulatory loop that dictates whether hepatic cells succumb to hyper-inflammatory injury or adapt through tolerogenic reprogramming [13,14,15]. Epigenetic gene silencing relies heavily on Polycomb Repressive Complexes (PRC1/PRC2) to maintain repressive histone marks and structural chromatin compaction [16,17]. Within this regulatory framework, Jumonji and AT-rich interaction domain-containing 2 (JARID2) acts as an essential gatekeeper by modulating PRC2 recruitment and enzymatic fidelity to fine-tune the transcriptional repressive landscape [18]. Consequently, the targeted downregulation of JARID2 destabilizes PRC-mediated repression, leading to a loss of local repressive marks and a structural opening of chromatin that allows previously silenced target genes to escape repression and undergo aberrant reactivation [19].

This repressive landscape is further complicated by the dynamic incorporation of histone variants. Histone H3.3, encoded by the *H3F3A* gene, is typically associated with transcriptionally active or poised chromatin states and is deposited independently of DNA replication [20]. Unlike canonical histone H3, which is incorporated strictly during the S-phase to package newly synthesized DNA, the replication-independent deposition of H3.3 allows for rapid, localized chromatin remodeling in post-mitotic or highly stressed cells [21]. The significance of H3F3A -encoded H3.3 lies in its ability to actively counter Polycomb-mediated repression; its enrichment at promoters and enhancers maintains an open, accessible chromatin architecture [22]. This prevents the spreading of repressive heterochromatin and keeps critical stress-responsive genes in a ‘poised’ state, ready for instantaneous transcription upon environmental cues.

To date, the exact mechanism linking exosome-mediated intercellular communication to localized hepatic chromatin remodeling during sepsis remains elusive. In this study, we bridge this gap by defining a novel regulatory axis wherein sepsis-induced exosomal *MAFB* mRNA drives hepatic miR-155 transactivation, leading to the dual post-transcriptional repression of the epigenetic gatekeeper *JARID2* and the histone variant *H3F3A*. Using a functional reconstruction model in hepatic cells, we demonstrate how this exosome-associated transcription factor axis triggers a “repressor-of-repressors” cascade, initiating a rapid transition from a homeostatic to a hyper-inflammatory state in the hepatic microenvironment.

## 2. Materials and Methods

### 2.1. Chemicals and Reagents

Unless otherwise specified, fine chemicals, solvents, and standard laboratory reagents were of analytical grade and purchased from Sigma-Aldrich (Merck KGaA, Darmstadt, Germany).

### 2.2. Cell Culture and In Vitro Sepsis Modeling

THP-1 Macrophage Culture and Stimulation: Human monocytic THP-1 cells (ATCC, Manassas, VA, USA) were maintained in OptiMEM medium (Thermo Fisher Scientific, Waltham, MA, USA). supplemented with 2% exosome-depleted fetal bovine serum (FBS), 2 mM L-glutamine at 37 °C in a humidified atmosphere with 5% CO_2_. To induce an in vitro model of endotoxemia/sepsis, THP-1 cells were differentiated into macrophages using phorbol 12-myristate 13-acetate (PMA—(Sigma-Aldrich, St. Louis, MO, USA)) and subsequently stimulated with lipopolysaccharide (LPS; *Escherichia coli* O111:B—(Sigma-Aldrich, St. Louis, MO, USA)) at a concentration of 100 ng/mL for 24 h. Control groups were treated with vehicle (PBS). Conditioned media were harvested for exosome isolation.

HepG2 Recipient Cell Culture: Human hepatoma HepG2 cells (ATCC, Manassas, VA, USA)—serving as an endogenous null-background system for MAFB and miR-155—were cultured in Dulbecco’s Modified Eagle Medium (DMEM) containing 10% FBS under standard conditions.

### 2.3. Exosome Isolation and Purification

Exosomes were isolated from the conditioned media of control and LPS-treated THP-1 macrophages using differential ultracentrifugation. Briefly, media were centrifuged at 300× *g* for 10 min, 2000× *g* for 20 min, and 10,000× *g* for 30 min at 4 °C to remove cells, dead cells, and cellular debris. The supernatant was then ultracentrifuged at 100,000× *g* for 70 min to pellet the raw vesicular fraction. The pellet was washed in sterile PBS and subjected to a final ultracentrifugation cycle at 100,000× *g* for 70 min. Purified exosome pellets were resuspended in PBS for immediate analysis or stored at −80 °C. Exosome yields were quantified across control and LPS-stimulated conditions (107 input donor cells per preparation) by measuring total vesicular protein concentration using the Qubit Protein Assay Kit on a Qubit Fluorometer (Thermo Fisher Scientific, Waltham, MA, USA) according to the manufacturer’s protocol.

### 2.4. Super-Resolution STED Microscopy Validation

For morphological and purity evaluation via Stimulated Emission Depletion (STED) microscopy, purified exosomes were immunolabeled using standard fluorescent lipid or canonical tetraspanin membrane dyes (Abberior Star Red—Abberior Instruments GmbH, Göttingen, Germany) for 30 min in the dark. Samples were then ultracentrifuged at 100,000× *g* for 70 min. Samples were pipetted onto coverslips and mounted with PROLONG mounting medium (Thermo Fisher Scientific, Waltham, MA, USA). Super-resolution imaging was executed utilizing an Abberior Instruments Expert Line STED microscope (Abberior Instruments GmbH, Göttingen, Germany, SN:SM181301) configuration equipped with appropriate depletion lasers at the Nano-Bio-Imaging Core Facility, University of Pécs, to resolve vesicular structures within the 30–150 nm range.

### 2.5. Lipidomic and Metabolite Profiling via MALDI-TOF MS

Extraction of lipids and metabolites was performed using a modified Bligh and Dyer method. Samples were resuspended in 150 mM ammonium acetate buffer (pH 7.4). A total of 160 μL of homogenate was transferred into a 1.5 mL microcentrifuge tube. Subsequently, 400 μL of HPLC-grade methanol (J.T. Baker, Phillipsburg, NJ, USA) was added, followed by vortexing. Then, 200 μL of analytical-grade chloroform (Carlo Erba, Milan, Italy) was added and the mixture was vortexed, resulting in a sample:methanol:chloroform ratio of 0.8:2:1. The mixture was incubated on ice for 10 min to allow extraction. After incubation, an additional 200 μL of chloroform and 200 μL of HPLC-grade water (Carlo Erba, Milan, Italy) were added. Samples were vortexed for 10 s and centrifuged at 20,000× *g* for 5 min at 4 °C. The resulting polar and non-polar phases were collected into separate HPLC glass vials and evaporated under a nitrogen stream at 40 °C until complete solvent removal. Dried samples were reconstituted in 50 μL of 60% methanol and stored until imaging. Two matrices were used for MALDI analysis: 9-aminoacridine (9-AA) for negative ion mode and 2,5-dihydroxybenzoic acid (DHB) for positive ion mode. Both matrices were prepared at a concentration of 10 mg/mL. DHB was dissolved in HPLC-grade methanol (J.T. Baker, Phillipsburg, NJ, USA) and stored at 4 °C. The 9-AA matrix was dissolved in a mixture of acetonitrile (J.T. Baker, Phillipsburg, NJ, USA) and isopropanol (Merck, Darmstadt, Germany) in a 40:60 ratio (*v*/*v*), using HPLC-grade solvents, and stored at 4 °C. Matrix application was performed as follows. Matrix and sample were mixed at a 1:1 ratio to a final volume of 6 μL per sample. From this mixture, 2 μL was spotted onto a polished steel target plate and dried rapidly under a forced air stream at room temperature. For calibration, red phosphorus was applied to a designated position on the plate for negative ion mode. For positive ion mode, a peptide calibration mix (Bruker, Billerica, MA, USA) was applied. Samples were analyzed in both positive and negative ion modes over a mass range of 500–2500 Da using an UltrafleXtreme MALDI-TOF/TOF mass spectrometer (Bruker, Billerica, MA, USA). Instrument settings were identical for both ion modes: digitizer set to 5.00 GS/s, Smartbeam laser set to medium, laser frequency at 2000 Hz, laser power at 90%, 200 laser shots per pixel, and a total of 15,000 shots accumulated per measurement. External calibration in positive ion mode was performed using Leucine-Enkephalin (Sigma-Aldrich, Saint Louis, MO, USA) and Bruker Peptide Calibration Mix (Bruker, Billerica, MA, USA). Negative ion mode calibration was conducted using red phosphorus clusters (Sigma-Aldrich, Saint Louis, MO, USA). Data acquisition and processing were performed using FlexAnalysis software (Bruker, Billerica, MA, USA). Peak detection was carried out using the SNAP algorithm with a signal-to-noise threshold of 6. Spectral smoothing was performed using the Savitzky–Golay algorithm (width 0.2 *m*/*z*, 1 cycle), and baseline subtraction was conducted using the TopHat method. Statistical analysis of spectral data was performed in R version 4.3.2. (R Foundation for Statistical Computing, Vienna, Austria) using the following packages: matrixTests, roperators, FELLA, KEGGREST, igraph, magrittr, and resample.

### 2.6. RNA Extraction and Next-Generation Sequencing (QuantSeq and Small RNA-Seq)

Co-isolation of RNA Species: Total intra-vesicular RNA, containing both long coding mRNAs and small non-coding RNAs, was isolated from identical exosomal preparations using a dual-retaining column methodology to prevent extraction bias (Direct-Zol microprep kit, Zymo Research, Irvine, CA, USA).

Transcriptomic Profiling (QuantSeq 3′ Tag-Seq): Coding transcripts were analyzed utilizing QuantSeq 3′ Tag-RNA-seq technology [23]. Libraries were generated from exosomal RNA replicates (Control *n* = 4; LPS-treated *n* = 3).

Parallel miRNA Sequencing [24]: Small RNA libraries were prepared concurrently to map the microRNA payload. All libraries were sequenced on an Illumina platform (NextSeq 550) at the Hungarian Centre for Genomics and Bioinformatics.

### 2.7. Bioinformatics, In Silico Modeling, and Functional Pathway Annotation

Differential Expression Analysis: Sequence reads were aligned, normalized, and analyzed for differential expression. Thresholds for significant transcript/miRNA packaging shifts were set at a Fold Change > 2 and *p* < 0.05.

Functional Annotation: The PANTHER (Protein Analysis THrough Evolutionary Relationships) Classification System was utilized to categorize significantly altered mRNA cargo based on biological functions and molecular pathways [25].

Transcription Factor and Target Prediction: Conserved MAFB consensus DNA-binding motifs were evaluated using JASPAR software [25]. MicroRNA-155 targets within the 3′ UTR of JARID2 and H3F3A were simulated and cross-referenced with GeneHacker (GH) interaction frameworks in the UCSC Genome Browser.

### 2.8. Plasmids, Cloning, and Transient Transfection

Human *MAFB* is a single-exon gene (RefSeq: NM_005461) and its complete coding sequence was directly amplified from human genomic DNA isolated from THP-1 cells using a nested PCR approach. Outer PCR: The initial amplification round utilized outer flanking primers targeting the 5′ and 3′ untranslated regions of the *MAFB* gene using a high-fidelity DNA polymerase (Q5^®^ High-Fidelity DNA Polymerase, New England Biolabs, Ipswich, MA, USA). A 1 ul aliquot of the diluted outer PCR product served as a template for the second-round (nested) reaction. All primer sequences used for quantitative PCR are listed in Supplementary Table S1. The inner nested primers contained 5′-terminal restriction site overhangs: a BamHI site was incorporated into the forward primer immediately preceding the *MAFB* start codon, and an EcoRI site was incorporated into the reverse primer downstream of the stop codon. The resulting nested amplicon was purified using a QIAquick Gel Extraction Kit (Qiagen, Hilden, Germany), double-digested with BamHI and EcoRI restriction endonucleases (New England Biolabs, Ipswich, MA, USA), and directionally ligated into the corresponding multiple cloning site of the digested pcDNA3.1(+) eukaryotic expression vector (Invitrogen/Thermo Fisher Scientific, Waltham, MA, USA) using T4 DNA Ligase. Recombinant clones (+MAFB) were transformed into competent *E. coli* DH5α cells, selected on ampicillin-containing agar, and validated by restriction enzyme digest and Sanger sequencing using standard T7 forward and BGH reverse sequencing primers to confirm sequence fidelity and correct reading frame orientation. Empty vector (+pcDNA3.1(+)) served as a negative control across all transfection experiments.

HepG2 recipient cells were seeded into plates and transiently transfected with either the *MAFB*-expressing plasmid (+MAFB) or the corresponding empty vector control (+pcDNA3) using GeneJuice reagents (Merck, Darmstadt, Germany) according to manufacturer protocols. Cells were harvested 48 h post-transfection for downstream analysis.

### 2.9. Quantitative Real-Time PCR (RT-qPCR) and microRNA Quantitation

mRNA Expression Analysis: For coding transcripts (*MAFB*, *JARID2*, *H3F3A*) and the long non-coding RNA host gene (*MIR155HG*), total RNA was reverse-transcribed into cDNA using random hexamer primers and ProtoScript^®^ II First Strand cDNA Synthesis Kit (New England Biolabs, Ipswich, MA, USA) according to the manufacturer’s protocol. Real-time PCR amplification was performed on a Bio-Rad CFX96 PCR machine (Bio-Rad Laboratories, Hercules, CA, USA) using SYBR Green master mix (Bio-Rad, Hercules, CA, USA). Relative expression levels were normalized to the endogenous control gene *ACTB* (Beta-actin). All primer sequences used for quantitative PCR are listed in Supplementary Table S1.

MicroRNA Quantitation via LNA Technology: For the specific quantification of mature *hsa-miR-155-5p*, reverse transcription and subsequent quantitative PCR were performed using the miRCURY LNA miRNA PCR Assay system (Qiagen, Hilden, Germany) to ensure high sensitivity and sequence-specific discrimination. Briefly, total or exosomal RNA was reverse-transcribed using the miRCURY LNA RT Kit (Qiagen, Hilden, Germany). The resulting cDNA was amplified using the target-specific miRCURY LNA miRNA PCR Assay primer set for *hsa-miR-155-5p*, combined with the miRCURY LNA SYBR Green PCR Kit (Qiagen, Hilden, Germany) on a real-time PCR cycler (CFX96). Normalization of mature miRNA levels was carried out using the recommended miRCURY LNA control assay (U6 small nuclear RNA) to account for technical variance.

PCR Data Processing: The relative fold-change in expression for both coding and non-coding targets was calculated using the comparative 2^ΔΔC^_T_ method. All reactions were performed in technical triplicates across at least three independent biological replicates.

### 2.10. Chromatin Immunoprecipitation (ChIP) Assay [26]

ChIP assays were performed in *MAFB*-transfected HepG2 cells. Chromatin was cross-linked with 1% formaldehyde, neutralized with glycine, and sonicated to shear DNA into fragments ranging from 200 to 500 bp. Immunoprecipitation was carried out overnight at 4 °C using a specific anti-MAFB antibody (R&D Systems, Minneapolis, MN, USA, NBP1-81342) or an isotype-matched control IgG (R&D Systems, Minneapolis, MN, USA NBP2-24891). Bound DNA complexes were eluted, cross-links were reversed, and purified DNA fragments were analyzed by qPCR using specialized primers targeting four candidate regulatory areas (Regions A, B, C, and D) across the *MIR155HG* locus (Supplementary Figure S1).

### 2.11. Electrophoretic Mobility Shift Assay (EMSA) [27]

To validate direct physical binding, recombinant human GST-tagged MAFB protein (Thermo Fisher Scientific, Waltham, MA, USA) was incubated with biotin-labeled double-stranded oligonucleotide probe matching the predicted B1 consensus binding sequence (5′-GTTCAAGAAACTGCTGAACACAC-3′) found within Region B of the *MIR155HG* promoter/enhancer. For competition assays, a 100-fold molar excess of unlabeled specific (5′-AACAAGAAACTGCTGACACACC-3′) competitor oligonucleotide was pre-incubated with the recombinant protein prior to adding the labeled probe. Protein-DNA complexes were separated via non-denaturing polyacrylamide gel electrophoresis and visualized using LightShift™ Chemiluminescent EMSA Kit (Thermo Fisher Scientific, Waltham, MA, USA)

### 2.12. Protein Extraction and Western Blotting [28]

HepG2 cells under designated experimental conditions (Control, +pcDNA3, +MAFB) were lysed in RIPA buffer [29] supplemented with protease and phosphatase inhibitor cocktails. Protein concentrations were determined using a Nanodrop. Equal amounts of total protein were resolved on SDS-PAGE gels and transferred to PVDF membranes (Thermo Fisher Scientific, Rockford, IL, USA). Membranes were blocked and incubated overnight at 4 °C with primary antibodies: anti-JARID2 (Thermo Fisher Scientific, Waltham, MA, USA, PA5-78031), anti-H3F3A (Thermo Fisher Scientific, Waltham, MA, USA, PA5-22388) and anti-ACTB (NB600-501—R&D systems, Minneapolis, MN, USA). Following incubation with appropriate HRP-conjugated secondary antibodies (Cell Signaling Technology, Danvers, MA, USA), protein bands were visualized using enhanced chemiluminescence (ECL—Thermo Fisher Scientific, Waltham, MA, USA) detection reagents H3F3A. Visualization was performed on a G-Box gel documentation system (Syngene, Synoptics, Cambridge, UK). Band intensity was quantified using ImageJ v1.54g (NIH, Bethesda, MD, USA). All experiments were performed in triplicate.

### 2.13. Statistical Analysis

All quantitative data are expressed as mean\pm standard deviation (SD) from at least three independent biological replicates. Comparative analyses between two distinct groups were evaluated using Student’s paired or unpaired *t*-test. For mass spectrometric and sequencing datasets, *p*-values were adjusted for multiple testing (*p*-adjusted) using False Discovery Rate (FDR) corrections. Statistical significance was defined as * *p* < 0.05 and ** *p* < 0.01.

### 2.14. Declaration of Generative AI in the Writing Process

During the preparation of this work, the authors used the Gemini AI tool in order to generate the conceptual schematic presented in Figure 7 After using this tool, the authors reviewed and edited the content as needed and take full responsibility for the content of the publication. The use of AI for text editing was strictly limited to improving grammar, spelling, and phrasing, which did not alter the original interpretation or meaning of the text.

## 3. Results

### 3.1. Verification of Exosome Isolation from LPS-Treated Samples Using STED Microscopy

**Figure 1:**
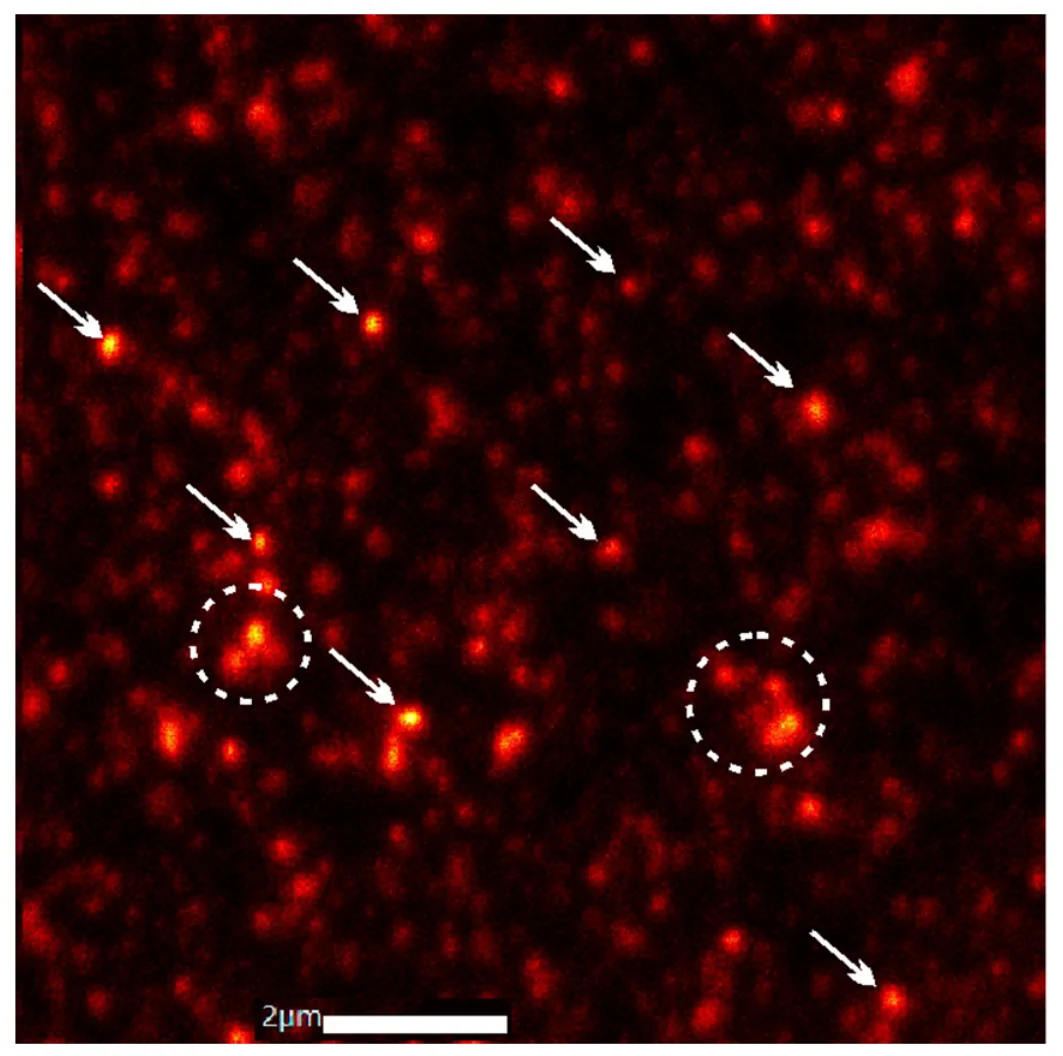
Visual validation of isolated exosomes via STED microscopy. Representative super-resolution STED image demonstrating the morphological profile of purified exosomes post-LPS stimulation (*n* = 3). Arrows indicate membrane particles (30–150 nm). Circles mark clusters resolved into individual vesicular components, confirming sample purity and the absence of significant amorphous debris. Scale bar = 2 µm.

Given that exosomes play a crucial role in intercellular communication by transferring functional mRNAs to recipient cells, we focused on isolating this specific vesicular population. To definitively confirm that the isolated membrane fraction consisted of intact nanoscale exosomes rather than cellular debris, we utilized super-resolution STED microscopy (Figure 1).

To characterize the impact of endotoxin exposure on exosome secretion, total vesicular yields were evaluated following isolation. Fluorometric Qubit analysis demonstrated that LPS stimulation induced a marked increase in exosomal release from THP-1 macrophages, yielding 2.4 ± 0.3 µg of total exosomal protein per 107 cells, compared to 0.75 ± 0.1 µg in unstimulated controls (*p* < 0.01; *n* = 3), representing a ~3.2-fold induction. Based on established biophysical benchmarks for density-gradient purified extracellular vesicles (>1010 particles µg of protein, this protein recovery corresponds to an estimated yield of ~2.4 × 1010 particles per 107 endotoxin-stimulated cells, confirming high-purity preparations suitable for downstream functional assays.

To model the downstream signaling events initiated upon exosome-mediated intercellular crosstalk, we established a functional reconstruction system in recipient HepG2 hepatocytes. Given that human HepG2 cells exhibit a clean, null-background profile lacking baseline endogenous *MAFB* and miR-155, this platform allowed us to isolate and map the precise transactivation and epigenetic disinhibition cascade triggered by ectopic *MAFB* expression without confounding cross-talk from host transcription factors.

### 3.2. Lipidomic Profiling of Isolated Membrane Fractions via MALDI-TOF MS

Complementing the structural and visual validation, we next characterized the molecular composition of the isolated membrane fraction using matrix-assisted laser desorption/ionization time-of-flight mass spectrometry (MALDI-TOF MS). This lipidomic analysis was conducted to profile the specific lipid signatures of the purified vesicles, further confirming their exosomal identity and structural preservation following LPS treatment (Table 1).

Mass spectrometric analysis indicated that the isolated membrane fractions from both the control and LPS-treated groups exhibited lipid profiles characteristic of canonical exosomes. The shared presence of these fundamental lipid clusters provides independent molecular validation that both experimental groups yielded highly purified vesicle populations.

However, comparative analysis revealed striking, statistically significant differences between the two exosome fractions, indicating robust lipidome remodeling upon endotoxin stimulation. LPS treatment was associated with a potential shift in the membrane profile, characterized by the apparent up-regulation of peaks tentatively identified as specific lipid species, alongside the concomitant suppression or absence of key control peaks (Table 2).

This pronounced architectural divergence suggests that beyond maintaining structural exosomal hallmarks, LPS activation actively alters the lipid landscape of the secreted vesicles, potentially impacting their downstream membrane fluidity and cellular target interactions. While characterizing the structural lipids and membrane-bound signaling molecules established the identity and purity of our exosome fractions, it also laid the groundwork for examining their transient intra-vesicular cargo.

### 3.3. Transcriptomic Profiling of Exosome-Encapsulated mRNA Cargo

**Figure 2:**
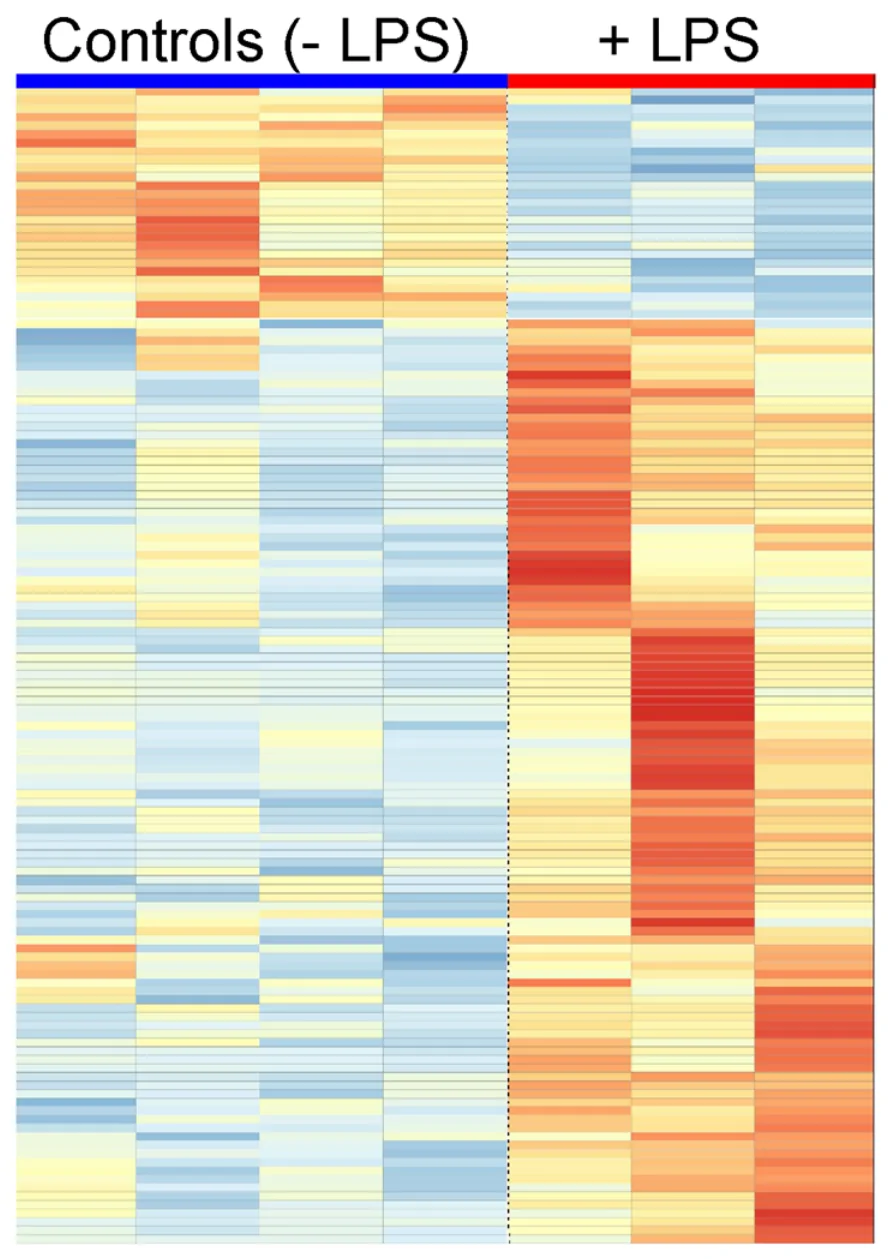
Hierarchical clustering analysis of exosome-encapsulated mRNA profiles. Heatmap depicting differentially packaged mRNA transcripts from control and LPS-treated THP-1 exosomal fractions obtained via QuantSeq 3′ Tag-RNA-seq (control *n* = 4 and LPS-treated *n* = 3 biological replicates per group). Relative expression levels are color-coded according to Z-score scale values, with red indicating enrichment and blue indicating depletion.

After establishing that LPS stimulation alters the lipid composition of the exosomal membrane, we next asked if this modification extended to the inner nucleic acid profiles. Therefore, we extracted the intra-vesicular RNA fractions from exosomes harvested from the conditioned media of both control and LPS-stimulated THP-1 cell cultures. These RNA samples were subsequently subjected to next-generation sequencing utilizing the QuantSeq technology, a very efficient 3′ Tag-Seq method. To evaluate shifts in the transcriptomic profiles encapsulated within the vesicles, the QuantSeq datasets from the control and LPS-treated exosomal fractions were compared using a hierarchical clustering heatmap approach (Figure 2). This global analysis revealed a stark divergence in the abundance of specific intra-vesicular transcripts between the two exosome fractions. Hierarchical clustering demonstrates tight intra-group reproducibility, segregating the biological replicates into clear, treatment-specific branches. Specifically, LPS challenge triggered a massive transcriptomic reprogramming event within the THP-1-derived vesicles.

This was characterized by the robust enrichment of a distinct cluster of pro-inflammatory, stress-responsive, and regulatory factor (i.e., transcription factor) encoding mRNAs, alongside the concomitant exclusion or depletion of transcripts typical of homeostatic control vesicles. QuantSeq analysis revealed a distinct shift in the exosomal mRNA profile of THP-1 cells following 24 h of LPS stimulation compared to untreated controls. A total of 136 mRNA transcripts were identified as significantly differentially packaged within the LPS-treated exosomal fractions (the complete annotated list of differentially expressed transcripts is provided in Supplementary Table S1). Among these, 109 mRNAs were enriched and 27 were downregulated (depleted) relative to the control group, applying a threshold of a fold change > 2 and a *p*-value < 0.05. This systematic restructuring of the encapsulated RNA profile underscores that endotoxin stimulation does not merely alter surface membrane characteristics, but actively modifies the internal transcriptional cargo intended for cellular communication.

### 3.4. Functional Classification of Differentially Packaged Exosomal mRNAs

To elucidate the molecular functions of the differentially packaged exosomal mRNAs, we performed a functional annotation analysis using the PANTHER Classification System [25]. This revealed that 48 genes (35% of the cargo) encode proteins heavily involved in biological regulation (Supplementary Table S2). Within this regulatory subset, PANTHER further classified 22 genes (Table 3) as key components implicated in transcriptional regulation, highlighting a targeted enrichment of gene expression-modulating cargo.

The selectively packaged exosomal cargo was enriched in critical epigenetic modifiers and chromatin-remodeling transcripts, including the histone deacetylase HDAC9 [30], the Polycomb Repressive Complex 1 core component RING1 [31], and the histone acetyltransferase complex subunit MCRS1 [32]. Alongside these DNA- and histone-modulating factors, we identified key epitranscriptomic regulators such as the m6A RNA-methylation adapter RBM15B [33]. The horizontal transfer of these specific epigenetic and chromatin-remodeling transcripts highlights a secondary layer of exosome-mediated communication, providing a direct mechanism for the long-range epigenetic reprogramming of recipient target cells under inflammatory stress. This specific exosome cargo displays an extensive transregulatory potential, capable of directly altering the transcriptional landscape of recipient target cells.

Among the heavily enriched transcription factor transcripts identified, the basic leucine zipper transcription factor MAFB stood out as a particularly compelling candidate due to its robust upregulation (Fold Change = 4.17, *p* < 0.001) and its established role in macrophage differentiation and inflammatory homeostasis [34]. Given its high abundance within the LPS-stimulated exosomal fraction and its known capacity to dictate myeloid gene expression networks, we selected MAFB for downstream functional validation to explore its specific transregulatory impact on recipient target cells.

### 3.5. Co-Packaged Regulatory Cargo: Characterization of the Parallel Exosomal miRNA Transcriptome

Because extracellular vesicles orchestrate intercellular communication through a complex set of multiple RNA species, evaluating the long-range regulatory capacity of these exosomes required a comprehensive mapping of their micro RNA content. Utilizing a specialized total RNA extraction methodology designed to isolate both long mRNAs and small non-coding RNAs, we profiled the microRNA landscape from the identical exosomal preparations. This parallel extraction approach effectively eliminated extraction bias and allowed us to directly cross-reference the enriched transcription factor transcripts with the concurrent miRNA profile co-packaged within the same stimulated vesicles (Table 4).

These parallel miRNA profiling data reveal that the exosomal cargo is not merely an assortment of isolated transcripts, but a highly coordinated, multi-layered regulatory payload. The synchronized packaging of key inflammatory miRNAs—such as the significantly enriched *hsa-miR-155-5p* and *hsa-miR-146a-5p*—complements the robust vesicular abundance of sequence-specific transcription factors identified in our coding mRNA dataset. Given that these co-packaged microRNAs are highly validated to target downstream epigenetic and signaling repressors, they likely function synergistically with the encapsulated coding transcripts to remodel the chromatin and transcriptional state of recipient cells. To decipher this functional cooperative network, we next sought to investigate the specific downstream regulatory gene cascade governed by *MAFB*, the most prominently upregulated transcription factor within this exosomal consortium.

### 3.6. Exogenous MAFB Overexpression Activates the MIR155HG Locus in a Null-Background Model

Elucidating the elements of the *MAFB*-governed regulatory network is of critical importance, as it may reveal how specialized exosomal cargo reprograms recipient cell transcription. A key prerequisite for mapping this gene-regulatory cascade was the selection of a host system has no background signals. To model the downstream signaling events initiated upon exosome-mediated intercellular crosstalk, we established a functional reconstruction system in recipient HepG2 hepatocytes. Given that human HepG2 cells exhibit a clean, null-background profile lacking baseline endogenous *MAFB* and miR-155, this platform allowed us to isolate and map the precise transactivation and epigenetic disinhibition cascade triggered by ectopic *MAFB* expression without confounding cross-talk from host transcription factors. This hepatic cellular model ensures that any subsequent alterations in the transcriptomic or epigenetic landscape can be attributed to the transfer or exogenous activation of the *MAFB*/*miR-155* axis. Notably, characterization of LPS-stimulated macrophage exosomes revealed that both *MAFB* mRNA and mature miR-155 are co-packaged as functional vesicular cargo. Consequently, direct incubation of recipient hepatocytes with whole exosomal fractions introduces an inherent confounding variable, making it difficult to deconvolute whether elevated intracellular mature miR-155 originates from direct exosomal RNA delivery or from de novo host transcription. To resolve this ambiguity and isolate the specific transactivation capacity of MAFB on host miR-155 biogenesis, we utilized our defined ectopic expression model.

**Figure 3:**
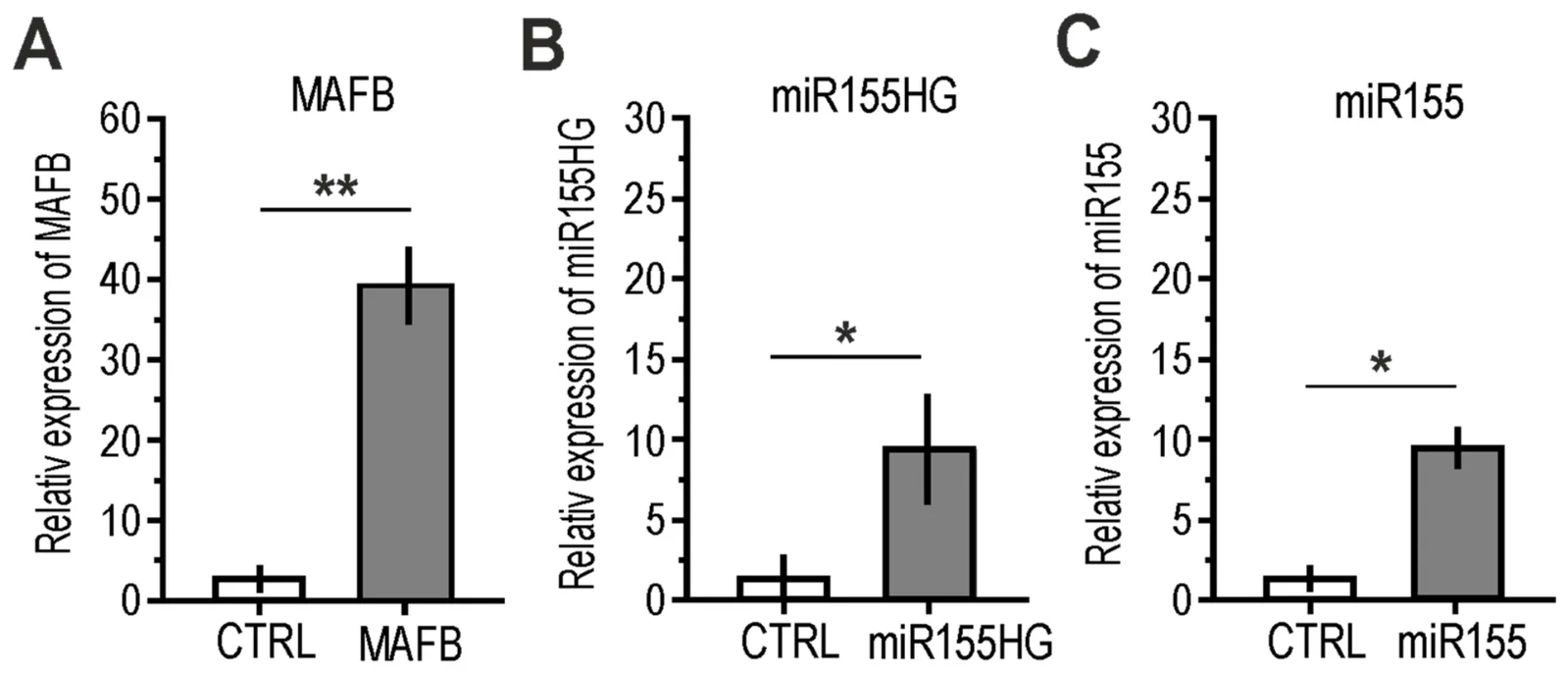
Transient overexpression of MAFB induces the upregulation of the *MIR155HG* locus and mature miR-155. Relative transcript expression levels were quantified via RT-qPCR in HepG2 cells 48 h post-transfection. (**A**) Validation of robust MAFB overexpression in cells transfected with the MAFB-expression plasmid compared to the empty vector control (CTRL). (**B**) Relative expression of the long non-coding RNA host gene *MIR155HG* following MAFB upregulation. (**C**) Relative expression level of mature miR-155-5p quantified using the miRCURY LNA miRNA PCR system. Data are presented as mean ± standard deviation (SD) from three independent biological replicates (*n* = 3). Statistical significance was determined using Student’s *t*-test; * *p* < 0.05, ** *p* < 0.01 compared to the respective control group.

To experimentally mimic MAFB transfer, we cloned the CDS of human MAFB into a eukaryotic expression vector, which was transiently transfected into HepG2 cells. Following transient transfection, RT-qPCR analysis confirmed a statistically significant upregulation of MAFB transcripts, which triggered a corresponding increase in expression of its downstream target, the *MIR155HG* host gene, as well as processed mature miR-155 (Figure 3).

Finally, to test whether this MAFB-driven JARID2/H3F3A suppression functionalizes chromatin reopening at PRC2-repressed loci, we queried public ENCODE ChIP-seq datasets in HepG2 cells. Analysis of the genomic serpin gene cluster revealed broad, continuous enrichment of the repressive H3K27me3 histone modification across the SERPINB2 locus (Figure S2), accounting for its completely null baseline expression in recipient hepatocytes. Following ectopic MAFB transfection, RT-qPCR analysis confirmed a robust de novo transcriptional induction of SERPINB2 (~6-fold increase vs. CTRL, *p* < 0.05; Supplementary Figure S2). These results demonstrate that MAFB-mediated disruption of the JARID2/PRC2 axis effectively relieves local H3K27me3-mediated silencing to license downstream gene transactivation.

By measuring both the primary host transcript (*MIR155HG*) and the mature microRNA, these results confirm that MAFB actively transactivates host endogenously transcribed miR-155, establishing a clear mechanistic framework for target activation distinct from direct exosomal microRNA transfer.

### 3.7. ChIP Analysis Reveals Direct MAFB Transactivation of the MIR155HG Locus via a Distal Enhancer-Promoter Loop

**Figure 4:**
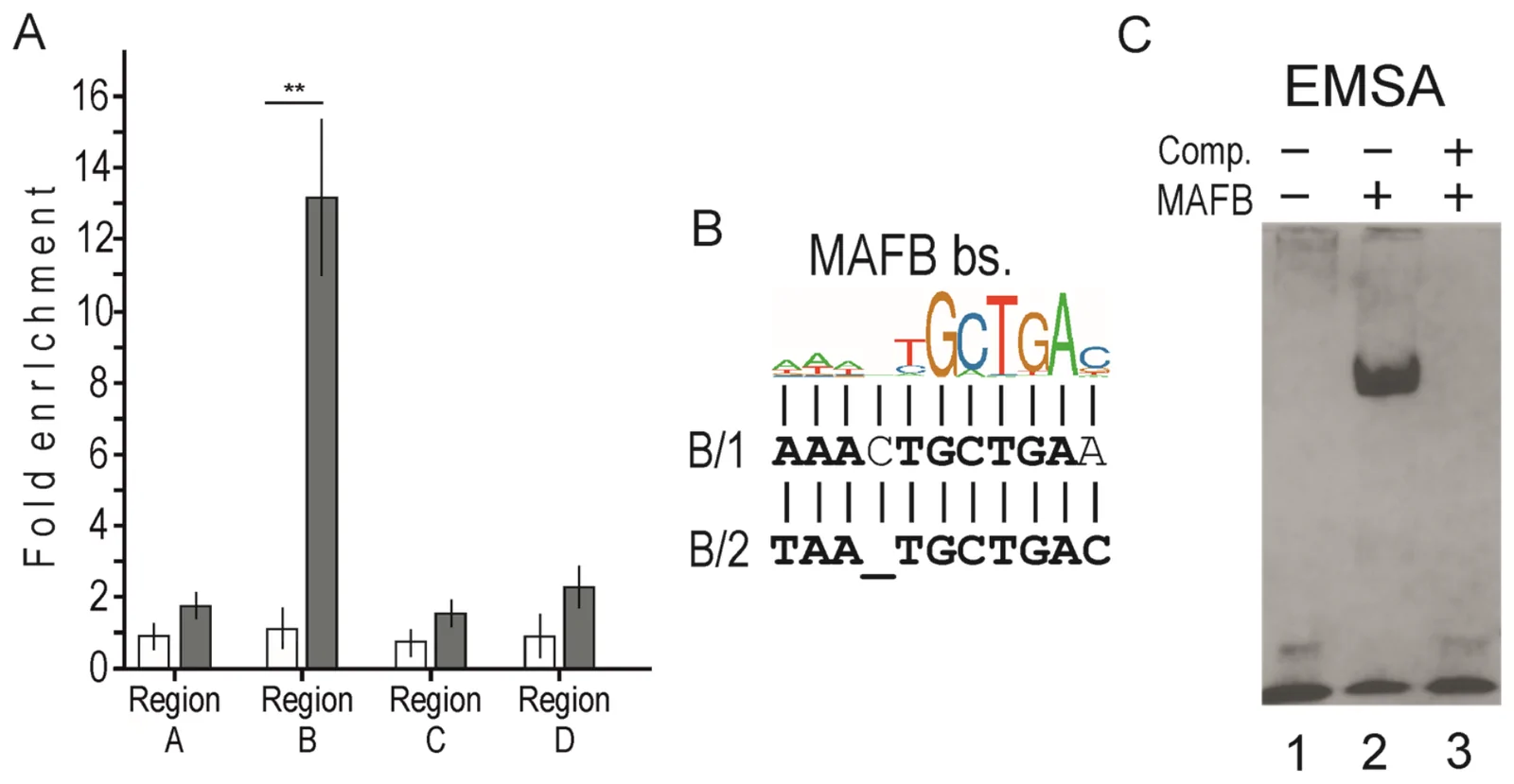
MAFB directly interacts with specific consensus binding sites within the regulatory region. (**A**) ChIP analysis evaluating MAFB enrichment across distinct genomic loci designated as Regions A, B, C, and D. Data are expressed as Fold Enrichment relative to control levels. A highly significant enrichment of MAFB is observed within Region B. (*n* = 3); ** *p* < 0.01). (**B**) Sequence alignment of the two identified consensus MAFB binding sites (B/1 and B/2) localized within Region B, mapped against the structural MAFB consensus binding logo sequence. (**C**) EMSA confirming direct physical interaction. Incubation of the labeled oligonucleotides spanning the Region B consensus motifs without recombinant MAFB protein served as the negative control (lane 1). Incubation of the labeled oligonucleotides with recombinant MAFB protein yielded a distinct high-molecular-weight DNA–protein complex (lane 2). The specificity of the interaction was demonstrated by the complete ablation of the shifted band upon the addition of an unlabeled specific competitor (lane 3).

To decipher the precise biochemical mechanism underlying this up-regulation, we investigated whether MAFB directly acts as a transcriptional activator of the MIR155HG locus or exerts its effects through indirect downstream signaling intermediaries. In silico analysis employing JASPAR software (release 11th), identified conserved MAFB consensus DNA-binding motifs distributed across the locus (Supplementary Figure S1). Cross-referencing these motifs with GeneHacker interaction data [35] downloaded from the UCSC Genome Browser allowed us to focus on four distinct high-probability regulatory target zones for mapping: three positioned upstream of the transcription start site and one located downstream within the gene body (Supplementary Materials Figure S1). To validate the physical occupancy of MAFB at these candidate regulatory architectures in vivo, ChIP assays targeting these four specific chromosomal regions were conducted in our transfected HepG2 cell model (Supplementary Materials Figure S1). Our ChIP-qPCR data demonstrated a significant enrichment of MAFB binding at region B compared to the IgG control, confirming direct physical interaction with the locus (Figure 4).

Region B harbors two predicted MAFB binding sites (Supplementary Figure S1). To verify the physical interaction of MAFB with these consensus motifs, we performed an EMSA using recombinant MAFB protein, which confirmed direct binding to both target sequences. Furthermore, consistent with the predictive UCSC interaction frameworks, chromatin mapping experiments revealed that a distal upstream enhancer B region physically interacts with the core *MIR155HG* promoter through a localized long-range chromatin looping event. These findings demonstrate that *MAFB* directly upregulates *MIR155HG* expression by anchoring to specialized regulatory elements and stabilizing a functional enhancer-promoter looping architecture.

### 3.8. The MAFB/miR-155 Axis Post-Transcriptionally Represses the Polycomb Cofactor JARID2

**Figure 5:**
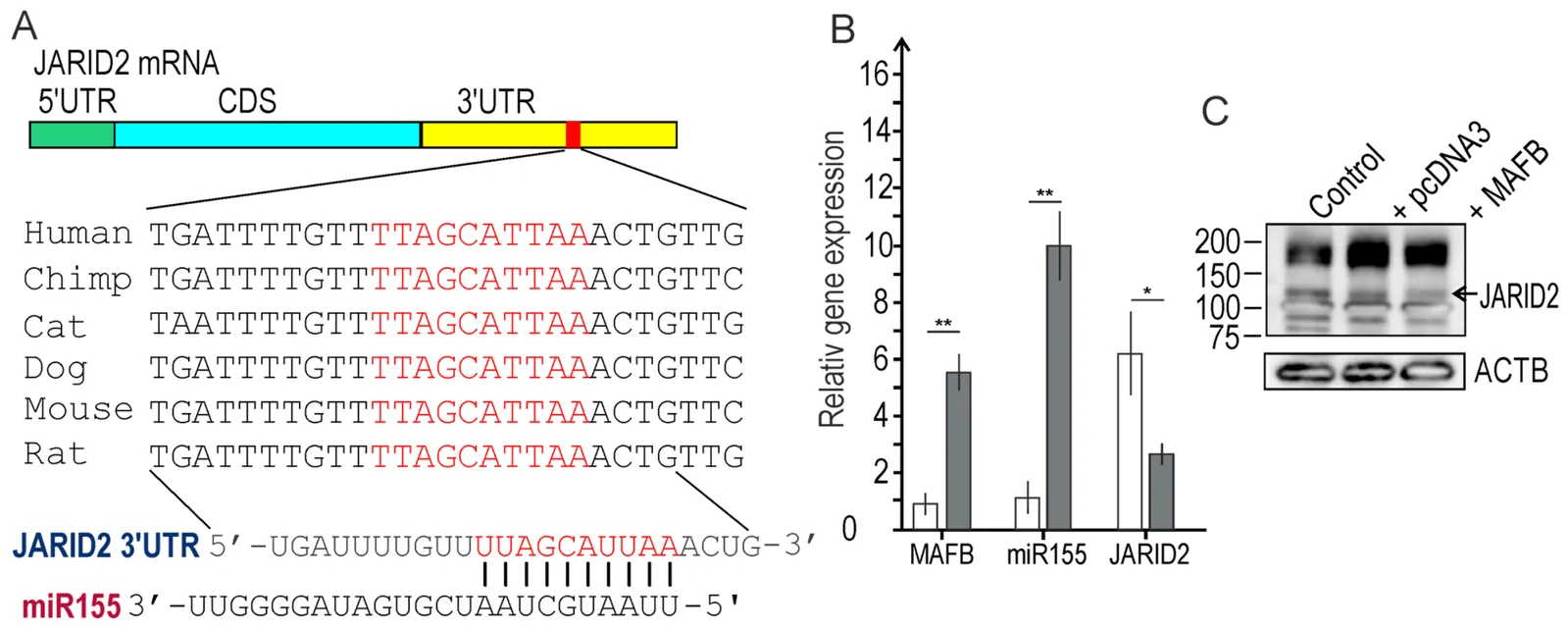
JARID2 is a direct post-transcriptional target of miR-155 modulated by MAFB. (**A**) Schematic representation of the *JARID2* mRNA transcript structure architecture, illustrating the 5′ UTR, coding sequence, and 3′ UTR. The red block denotes a highly conserved miR-155 complementary target site located within the 3′ UTR. Interspecies sequence alignment (Human, Chimp, Cat, Dog, Mouse, and Rat) demonstrates the evolutionary conservation of the seed region (red). The predicted base-pairing interactions between the *JARID2* 3′ UTR binding sequence and the mature human miR-155 sequence are shown below, with vertical lines indicating canonical Watson–Crick seed complementary binding. (**B**) Quantitative real-time PCR (RT-qPCR) analysis of *MAFB*, *miR-155*, and *JARID2* transcript levels under control (white bars) versus experimental treatment/overexpression conditions (gray bars). Data are normalized and expressed as relative gene expression. Overexpression of the upstream cascade elements correlates with a reciprocal knockdown/suppression of downstream endogenous *JARID2* transcripts. Data represent mean SD (*n* = 3); * *p* < 0.05, ** *p* < 0.01. (**C**) Western blot analysis of endogenous JARID2 protein levels following transient transfection. Cell lysates from untreated control, empty vector control (+pcDNA3), and MAFB-overexpressing (+MAFB) conditions were immunoblotted using a specific anti-JARID2 antibody. Beta-actin (ACTB) was utilized as the internal loading control. Molecular weight markers (kDa) are indicated on the left. Overexpression of MAFB leads to a visible decrease in endogenous JARID2 protein abundance.

Having established that *MAFB* directly binds and trans-activates the *MIR155HG* genomic locus, we next sought to determine whether this newly upregulated microRNA payload functionally represses its downstream epigenetic targets in our hepatic model. A primary candidate node within this cascade is JARID2, a critical regulatory cofactor of the PRC2 known to maintain repressive chromatin states. To evaluate the downstream impact of the *MAFB*/*miR-155* activation cascade on *JARID2* expression, we analyzed both transcript and protein levels in transiently transfected HepG2 cells (Figure 5).

RT-qPCR analysis revealed a significant decrease in *JARID2* mRNA abundance following *MAFB* overexpression compared to empty-vector controls (Figure 5B), indicating transcript destabilization. To confirm whether this reduction extended to the functional gene product, we performed Western blot analysis to track protein expression dynamics (Figure 5C). In alignment with our transcriptomic data, we detected downregulation of JARID2 at the protein level. (Figure 5C)

These data demonstrate that the *MAFB*-driven induction of *miR-155* exerts strong post-transcriptional and translational repression on *JARID2*, effectively breaching a major epigenetic gatekeeper in recipient cells.

### 3.9. The MAFB/miR-155 Axis Targets the Histone Variant H3F3A to Induce Core Chromatin Remodeling

**Figure 6:**
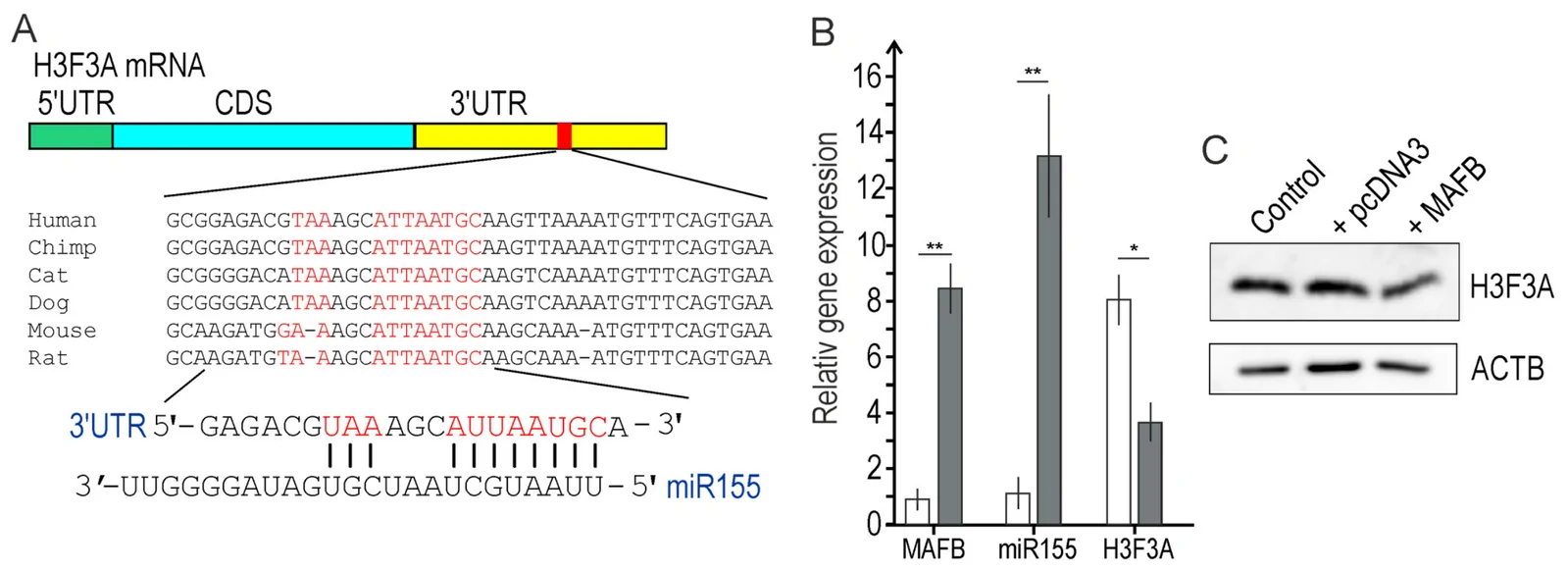
*H3F3A* is a direct post-transcriptional target of miR-155 modulated by MAFB. (**A**) Schematic representation of the *H3F3A* mRNA transcript architecture, illustrating the 5′ UTR, coding sequence, and 3′ UTR. The red block denotes a highly conserved miR-155 complementary target site located within the 3′ UTR. Interspecies sequence alignment (Human, Chimp, Cat, Dog, Mouse, and Rat) demonstrates the evolutionary conservation of the critical target and seed regions (red text). The predicted base-pairing interactions between the *H3F3A* 3′ UTR binding sequence and the mature human miR-155 sequence are displayed below, with vertical lines indicating canonical Watson–Crick complementary seed binding. (**B**) RT-qPCR analysis of *MAFB*, *miR-155*, and *H3F3A* transcript levels under control (white bars) versus experimental treatment/overexpression conditions (gray bars). Data are normalized and expressed as relative gene expression. Overexpression of the upstream cascade elements correlates with a reciprocal downregulation and knockdown of endogenous *H3F3A* transcripts. Data represent mean SD (*n* = 3); * *p* < 0.05, ** *p* < 0.01. (**C**) Western blot analysis of endogenous H3F3A protein levels following transient transfection. Cell lysates from untreated control, empty vector control (+pcDNA3), and MAFB-overexpressing (+MAFB) conditions were immunoblotted using a specific anti-H3F3A antibody. Beta-actin (ACTB) was utilized as the internal loading control. Overexpression of MAFB results in a distinct reduction in endogenous H3F3A protein expression.

Having demonstrated that the *MAFB*/*miR-155* pathway efficiently compromises the Polycomb repressive cofactor *JARID2*, we next addressed whether this localized epigenetic remodeling is reinforced by the parallel depletion of fundamental structural histone variants. While silencing enzymatic modifiers alters downstream chromatin-writing activity, achieving complete and sustained transcriptional reprogramming often requires a concurrent disruption to the physical nucleosome core itself. To explore this potential two-pronged epigenetic assault, we targeted H3F3A, an essential gene encoding the replication-independent histone variant H3.3 that is classically responsible for maintaining open, transcriptionally permissive euchromatin domains. Our in silico sequence alignments and microRNA-target interaction models identified highly conserved, high-affinity binding configurations for *miR-155* within the 3′ untranslated region (3′UTR) of the *H3F3A* transcript (Figure 6), positioning it as a prime candidate for direct post-transcriptional restriction.

Our biochemical and expression data confirm that H3F3A is a direct downstream target of the MAFB/miR-155 regulatory cascade. This targeted depletion of a primary replication-independent histone variant provides a clear molecular mechanism for the rapid destabilization of homeostatic chromatin domains, effectively priming the host cell for sustained inflammatory gene expression.

## 4. Discussion

The clinical progression of sepsis-induced acute liver injury is largely driven by a profound, dysregulated communication network operating between activated innate immune cells and the hepatic parenchyma. While traditional paradigms have heavily focused on the endocrine-like actions of circulating pro-inflammatory cytokines (e.g., TNF-α, IL-6), these pathways often rely on classic receptor–ligand configurations that trigger predictable intracellular cascades [36]. In the present study, we define an alternative axis of remote organ reprogramming characterized by sepsis-induced modifications in exosomal transcription factor mRNA cargo. Specifically, our data elucidate a sequential pathway wherein LPS-activated macrophages enrich functional MAFB mRNA payloads into exosomes. To model the downstream consequences of this intercellular axis in hepatic cells, plasmid-mediated overexpression of MAFB in a null-background HepG2 host system demonstrated that ectopic MAFB directly transactivates the MIR155HG locus, executing a robust “repressor-of-repressors” disinhibition cascade by simultaneously repressing the epigenetic modifiers JARID2 and H3F3A.

Our mass spectrometric and structural validation confirmed that endotoxin stimulation significantly remodels both the lipid bilayer architecture and the internal cargo profile of THP-1-derived vesicles. The distinct enrichment of sphingosine 1-phosphate (S1P) exclusively within the LPS-stimulated exosomal fraction (Table 2) is particularly notable, as S1P is a well-documented bioactive lipid mediator involved in endothelial barrier destabilization and directed immune cell trafficking [37,38].

Crucially, our QuantSeq 3′ Tag-RNA-seq analysis revealed that the intra-vesicular RNA pool is not an arbitrary reflection of the dying macrophage’s cytoplasm, but a highly curated regulatory payload. Out of 136 significantly shifted transcripts, PANTHER classification identified a striking enrichment of sequence-specific transcription factors, led by a 4.17-fold increase in MAFB mRNA. While multiple transcription factor-encoding mRNAs were robustly enriched within the endotoxin-stimulated vesicular cargo, we specifically prioritized MAFB for downstream functional modeling due to a critical physiological constraint of our recipient host system. Under baseline homeostatic conditions, human HepG2 hepatoma cells exhibit an entirely null expression profile for both endogenous MAFB and its downstream effector miR-155. Selecting a candidate transcription factor with zero background expression in the recipient system was experimentally paramount; it provided an exceptionally clean, baseline-absent genetic environment. This strategic configuration guarantees that any subsequent transactivation events, microRNA maturations, or downstream chromatin-remodeling signatures observed post-expression can be unequivocally attributed to ectopic MAFB, completely eliminating the confounding variables of cross-talk or synergy with pre-existing host transcription factors.

The choice of MAFB as a primary candidate node reveals an intriguing biological paradox. Endogenous MAFB typically serves as a critical anti-inflammatory checkpoint in mature myeloid lines, keeping pro-inflammatory cytokine expression restricted. However, its targeted horizontal transport into hepatocytes serves an entirely different, trans-organ regulatory purpose. By utilizing this clean, null-background HepG2 expression model, we established that ectopic hepatic MAFB expression is a powerful upstream driver of the MIR155HG locus. Our ChIP-qPCR and EMSA validation across Region B successfully demonstrated that MAFB directly anchors to specialized consensus motifs within a distal upstream enhancer element. This physical binding event coordinates a localized, long-range chromatin looping architecture that stabilizes contact with the core MIR155HG promoter, triggering a robust induction of mature miR-155. This parallel activation mechanism perfectly mirrors our small RNA sequencing data (Table 4), where mature hsa-miR-155-5p was found to be heavily co-packaged alongside its upstream MAFB activator within the identical exosomal preparations.

This co-delivery architecture suggests a multi-layered mechanism designed to ensure sustained activation of this inflammatory pathway in recipient hepatic cells. The directly transferred, exosome-derived miR-155 pool is capable of acting transiently and immediately upon recipient cell entry. This pre-packaged miR-155 bridges the time gap required for ectopic MAFB mRNA to undergo translation, nuclear translocation, and subsequent transactivation of the host cell’s endogenous MIR155HG locus. By the time this endogenous transcriptional machinery is fully operational and generating sustained levels of host miR-155, the initial exosomal miR-155 payload has already initiated the silencing cascade.

Once elevated in recipient hepatocytes, mature miR-155 exerts a coordinated, dual-action post-transcriptional assault on the host cell’s baseline epigenetic architecture. Rather than focusing on isolated microRNA targets, our findings demonstrate a concurrent suppression of the PRC2 auxiliary recruiter JARID2 and the structural histone variant H3F3A. Under homeostatic conditions, JARID2 preserves PRC2-mediated H3K27me3 repression [39,40], while H3.3 prevents heterochromatin spreading. In public ENCODE ChIP-seq datasets in baseline HepG2 cells, the locus containing the genomic serpin cluster—including SERPINB2—is heavily decorated by repressive H3K27me3 marks and occupied by PRC2 core components (EZH2 and SUZ12), maintaining it in a tightly silenced heterochromatic state. Because JARID2 serves as a pivotal recruiter and activator of the PRC2 complex, its miR-155-mediated post-transcriptional suppression disrupts H3K27me3 catalysis and local heterochromatin compaction. In our setup, this “repressor-of-repressors” cascade leads directly to the derepression and de novo transcriptional initiation of SERPINB2 upon ectopic MAFB expression.

By simultaneously suppressing JARID2 and H3F3A at both transcript and protein levels (Figure 4 and Figure 5), the MAFB/miR-155 axis effectively breaches baseline transcriptional containment. The concurrent ablation of a core enzymatic recruiter and a key structural nucleosomal variant triggers a rapid collapse of local heterochromatin compaction, transitioning closed, repressive domains into an open, accessible euchromatin state. To establish SERPINB2 as a functional exemplar readout of this derepression axis without expanding the manuscript’s scope into comprehensive in vivo profiling, we confirmed that this baseline-silent gene is robustly induced following MAFB transactivation Fsuppl. This structural reopening licenses the immediate, unhindered transactivation of downstream hepatic stress targets and acute-phase response genes responsible for the clinical manifestations of severe endotoxemia, including acute inflammation, metabolic dysfunction, and coagulopathy.

A clear limitation of the current study lies in the experimental distinction between exosomal cargo loading and direct proof of functional exosomal translation in recipient cells. While recipient cell exosomal uptake and cargo enrichment were experimentally validated, our mechanistic downstream dissection relied on plasmid-based MAFB transfection in HepG2 cells. This approach was intentionally selected to isolate the transactivation and epigenetic cascade in a clean, zero-background environment without interference from endogenous transcripts. However, it serves as a functional reconstruction model of downstream signaling rather than direct, quantitative tracking of endogenously delivered exosomal MAFB mRNA translation. Future studies employing live-cell single-molecule translation reporters or bio-orthogonal tagging in primary human hepatocyte systems will be essential to precisely quantify the translation kinetics of naturally transferred exosomal mRNA payloads in vivo.

**Figure 7:**
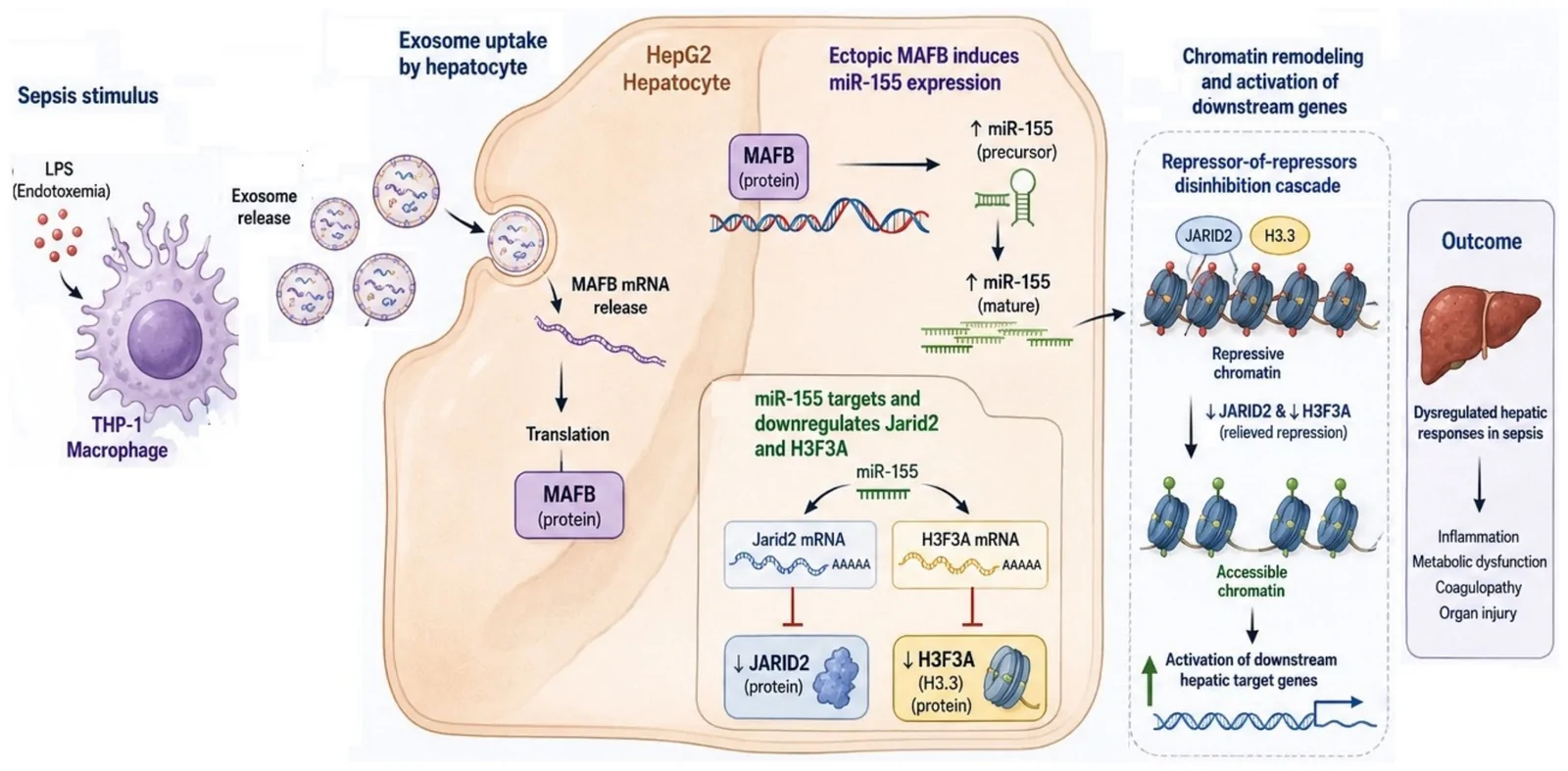
Proposed mechanistic model of macrophage-derived exosomal *MAFB* signaling and downstream miR-155-mediated chromatin remodeling in hepatic cells. LPS-stimulated macrophage exosomes are enriched with *MAFB* mRNA cargo and internalized by recipient hepatocytes. To functionally reconstruct downstream signaling in recipient cells, ectopic *MAFB* expression directly transactivates miR-155 maturation. Elevated miR-155 directly downregulates the Polycomb cofactor *JARID2* and the structural histone variant *H3F3A* (encoding H3.3). This simultaneous post-transcriptional silencing remodels closed, repressive chromatin into an accessible state, licensing the activation of downstream hepatic target genes responsible for sepsis-induced inflammation, metabolic dysfunction, and organ injury.

In conclusion, this study uncovers an intricate immune-hepatic trans-regulatory axis that shifts the focus from simple cytokine signaling to complex, long-range epigenetic reprogramming. This sequential exosomal MAFB → miR-155↑ → JARID2↓ and H3F3A↓ pathway provides concrete, molecular checkpoints for therapeutic intervention. Future strategies designed to interrupt the exosomal encapsulation of MAFB mRNA, inhibit the specific enhancer-promoter loop regulating MIR155HG, or synthetically stabilize JARID2/H3.3 interactions may yield novel therapeutic avenues to combat sepsis-induced liver injury and systemic organ failure (Figure 7).

## 5. Conclusions

In this study, we identified a novel, exosome-mediated immune-hepatic crosstalk mechanism associated with epigenetic remodeling during acute endotoxemia. We demonstrated that LPS-stimulated macrophages selectively package functional *MAFB* mRNA into circulating exosomes that are internalized by hepatic cells. Using a functional reconstruction model in null-background HepG2 hepatocytes, ectopic expression of this transcription factor directly binds the *MIR155HG* enhancer (Region B) to drive mature miR-155 expression. This localized induction of miR-155 executes a highly coordinated “repressor-of-repressors” disinhibition cascade, simultaneously downregulating the enzymatic PRC2 component *JARID2* and the structural histone variant *H3F3A* (encoding H3.3). The resulting collapse of homeostatic heterochromatin compaction licenses the unchecked transcription of downstream inflammatory and metabolic genes, culminating in acute liver injury. Ultimately, uncovering this sequential exosomal *MAFB* cargo enrichment exosomal MAFB → miR-155↑ → JARID2↓ and H3F3A↓ axis provides precise molecular checkpoints for the development of targeted therapeutic interventions to mitigate sepsis-induced organ failure.

## Figures and Tables

**Table 1:** Differential metabolite profiling and exosomal relevance under control and LPS-stimulated conditions. The 10 top tentatively identified metabolic assignments across Control and LPS-treated groups. For each condition, the unique KEGG identifiers, chemical assignments, and adjusted *p*-values (*p*-adj) are provided alongside their documented biochemical or structural relevance to exosomal membranes and cargo packaging. FMN, riboflavin-5-phosphate; HODDA, 2-hydroxy-6-oxonona-2,4-diene-1,9-dioate; KEGG, Kyoto Encyclopedia of Genes and Genomes; LPS, lipopolysaccharide; SFA, saturated fatty acid.

Exosomes	KEGG	Identified Assignment	*p*-adj	Exosome-Specific Relevance
**Control**	C18044	Pregnenolone sulfate	5.0 × 10^−6^	Enriched in exosome membrane
	C00061	FMN; Riboflavin-5-phophate	4.3 × 10^−5^	Soluble metabolic cofactor
	C01120	Sphinganine 1-phosphate	8.2 × 10^−5^	**Canonical Exosome Marker**
	C00468	Estrone	8.3 × 10^−5^	Hydrophobic steroid molecule
	C00013	Diphosphate/Pyrophosphate	8.7 × 10^−5^	Inorganic phosphate
	C04479	HODDA	3.1 × 10^−4^	Metabolic clearing product
	C08281	Docosanoic acid (Behenic acid)	4.1 × 10^−4^	LC-SFAs are highly enriched in Exos.
	C06425	Icosanoic acid (Arachidic acid)	4.9 × 10^−4^	Exosomal membrane integrity
	C14794	2,3-Dinor-8-iso prostaglandin F2α	5.2 × 10^−4^	Eicosanoid signaling derivative
	C00365	dUMP; Deoxyuridylic acid	3.0 × 10^−6^	Reflects nucleic acid packaging
**LPS-treated**	C18044	Pregnenolone sulfate	3.0 × 10^−6^	Conserved sterol framework
	C01120	Sphinganine 1-phosphate	6.2 × 10^−5^	**Canonical Exosome Marker**
	C00517	Hexadecanal/Palmitaldehyde	6.4 × 10^−5^	Sphingolipid Breakdown Product
	C00249	Hexadecanoic acid (Palmitic acid)	7.3 × 10^−5^	Fatty acid anchor in exosome
	C01571	Decanoic acid (Capric acid)	8.5 × 10^−5^	Modulates membrane fluidity
	C00013	Diphosphate/Pyrophosphate	1.7 × 10^−4^	Inorganic phosphate
	C01613	Stachyose	2.2 × 10^−4^	Oligosaccharide
	C14794	2,3-Dinor-8-iso prostaglandin F2α	2.5 × 10^−4^	Bioactive Lipid Cargo

**Table 2:** Distinct molecular cargo characterizing treatment-specific exosomal fractions. The top 5 most statistically significant lipid and metabolite species resolved exclusively within either the LPS-treated or Control primary hepatic exosome fractions via MALDI-TOF MS analysis. Features represent complete phenotypic separation, where baseline homeostatic components are either entirely exhausted or immunologically reprogrammed into signaling payloads following endotoxin challenge. Data represent adjusted *p*-values (*p*-adjust.) calculated by relative peak intensity variance thresholds. dUMP: Deoxyuridylic acid; dIDP: 2′-Deoxyinosine-5′-diphosphate.

Exosomes	KEGG ID	Identified Assignment	*p*-Adjust.	Exosome-Specific Relevance
**LPS-treated**	C00365	dUMP; Deoxyuridylic acid	3.0 × 10^−6^	Nucleotide cargo alteration
	C01571	Decanoic acid (Capric acid)	8.5 × 10^−5^	Cellular inflammatory response
	C01613	Stachyose	2.2 × 10^−4^	Oligosaccharide
	C11131	2-Methoxyestradiol 3-glucuronide	5.4 × 10^−4^	Conjugated steroid cargo
	C06124	Sphingosine 1-phosphate (S1P)	3.9 × 10^−3^	High-Impact Exosome Marker
**Control**	C06188	Salicin 6-phosphate	5.0 × 10^−6^	Baseline cell-culture metabolite
	C01344	dIDP	7.5 × 10^−4^	Nucleoside phosphate
	C18044	Pregnenolone sulfate	1.2 × 10^−3^	Lipid Raft Component
	C11133	Estrone 3-glucuronide	1.3 × 10^−3^	Baseline steroid cargo
	C03852	Androstan-3alpha,17beta-diol	2.0 × 10^−3^	Baseline steroid cargo

**Table 3:** Differential packaging of transcription factors and epigenetic regulators in THP-1-derived exosomes following LPS stimulation. List of significantly altered exosomal mRNA cargo identified by QuantSeq analysis following a 24 h lipopolysaccharide (LPS) challenge (*p* < 0.05). Positive fold change (FC) values represent transcripts enriched in the LPS-treated group, while negative values indicate transcripts depleted relative to untreated controls. Descriptions highlight the structural classification of sequence-specific transcription factors (TFs) alongside key chromatin-remodeling and epigenetic factors (e.g., *RING1*, *HDAC9*, *MCRS1*, and *RBM15B*).

Gene	Name	FC	*p* val.	Description
***BARX2***	Homeobox protein BarH-like 2	3.78	0.03	Homeodomain TF
***CREB3***	cAMP-responsive element-binding protein 3	6.48	0.04	Basic leucine zipper TF
***ZNF33B***	Zinc finger protein 33B	6.18	0.00	C2H2 zinc finger TF
***TSHZ2***	Teashirt homolog 2	6.13	0.00	Zinc finger TF
***BCL6***	B-cell lymphoma 6 protein	5.97	0.05	C2H2 zinc finger TF
***ZMIZ2***	Zinc finger MIZ domain-containing protein 2	5.04	0.01	Ubiquitin-protein ligase
***MAFB***	**Transcription factor MafB**	**4.17**	**0.001**	**Basic leucine zipper TF**
***MTF1***	Metal regulatory transcription	3.45	0.01	C2H2 zinc finger TF
***ZNF600***	Zinc finger protein 600	3.03	0.01	C2H2 zinc finger TF
***BHLHE40***	Class E basic helix-loop-helix	2.91	0.02	Basic helix-loop-helix TF
***IRF7***	Interferon regulatory factor 7	2.91	0.04	Winged helix/forkhead TF
***TFE3***	Transcription factor E3	2.52	0.05	Basic helix-loop-helix TF
***JUNB***	Transcription factor JunB	2.42	0.05	Basic leucine zipper TF
***IRX3***	Iroquois-class homeodomain	−2.22	0.04	Homeodomain TF
***RING1***	Ring Finger Protein 1	−2.43	0.02	PRC 1
***TNRC6B***	trinucleotide repeat containing 6B	−2.47	0.04	Component of the RISC
***HDAC9***	Histone Deacetylase 9	−3.00	0.03	Epigenetic factor
***MCRS1***	microspherule protein 1	−3.05	0.04	Epigenetic factor
***GFI1***	Zinc finger protein Gfi-1	−3.08	0.03	C2H2 zinc finger TF
***ZNF350***	Zinc finger protein 350	−3.12	0.001	C2H2 zinc finger TF
***RBM15B***	RNA binding motif protein 15B	−3.50	0.03	Adapter in m6A MT complex
***UBTF***	Upstream Binding Transcription Factor	−4.34	0.02	TF for rRNA

**Table 4:** Differential expression profile and functional targets of co-isolated exosomal microRNAs following LPS stimulation. Altered miRNA cargo isolated concurrently alongside mRNA fractions from THP-1-derived exosomes after 24 h of LPS exposure (*p* < 0.05). Fold change values represent miRNAs enriched in the endotoxin-stimulated exosomal payload, led by the highly upregulated *hsa-miR-1282*, *hsa-miR-181d-5p*, and *hsa-miR-584-5p*. Target genes represent experimentally validated downstream effectors known to regulate chromatin dynamics (*JARID2*, *DNMT1*), innate immune feedback loops (*SHIP1*, *SOCS1*, *TRAF6*), and cellular metabolic shifts (*HIF1A*).

miRNA ID	Fold Change	*p*-Value	Validated Target Gene(s)
*hsa-miR-1282*	15.27	0.032	***FOCAD*, *EIF4EBP1***
*hsa-miR-181d-5p*	9.43	0.010	***MAPK1*, *BCL2***
*hsa-miR-584-5p*	9.29	0.021	***ROCK1*, *YAP1***
*hsa-miR-30c-1-3p*	7.99	0.042	***BIRC6*, *ATG5***
*hsa-miR-146a-5p*	2.67	0.006	***TRAF6*, *IRAK1***
*hsa-miR-340-5p*	2.57	0.018	***MITF*, *IL-6R***
*hsa-miR-155-5p*	2.31	0.003	***JARID2*, *SHIP1*, *SOCS1***
*hsa-miR-210-3p*	2.22	0.009	***HIF1A*, *ISCU***
*hsa-miR-148a-3p*	1.82	0.047	***DNMT1*, *IKBKB***
*hsa-let-7d-5p*	−1.88	0.037	***HMGA2*, *TLR4***
*hsa-miR-92b-5p*	−2.35	0.039	***TSC1*, *RECK***
*hsa-miR-25-5p*	−4.79	0.034	***KLF4*, *BIM***

## Data Availability

All data reported in the paper will be shared by the corresponding author upon request.

## Supplementary Materials

The following supporting information can be downloaded at: https://www.mdpi.com/article/10.3390/cells15161481/s1, Figure S1: Genomic architecture and targeted ChIP-qPCR regions within the human *MIR155HG* locus; Figure S2: Baseline epigenetic silencing of *SERPINB2* by H3K27me3 and its *de novo* transcriptional induction following *MAFB* expression in HepG2 cells. Table S1: Differentially enriched mRNAs; Table S2: Primers for cloning and RT-qPCR.
