# Supplementary material for: Sepsis-Induced Exosomal Transfer of MAFB mRNA Reprograms Hepatocytes via a miR-155–Jarid2–H3F3A Epigenetic Cascade
Source: Cells. 2026 Aug 18;15(16):1481. doi: 10.3390/cells15161481 (PMC13511736; doi:10.3390/cells15161481)
Supplement: Supplementary file 1 [file cells-15-01481-s001.zip › Supplementary Material Figures.pdf]

Supplementary material

Sepsis-Induced Exosomal Transfer of *MAFB* mRNA Reprograms Hepatocytes via a miR-155–*Jarid2–H3F3A* Epigenetic Cascade

Gizaw Mamo Gebeyehu [1], Milorad Zjalic [2], Rita Bognár [1], Benjámin Farkas [1], Shima Rashidiani [3], Géza Makkai [4], Tibor Z. Jánosi [4], Péter Urbán [5], József Kun [5], Attila Gyenesei [5], Marianna Pap [1,6], Željko Debeljak [7,8], Marija Heffer [2] and Tibor A. Rauch [1,*]

1 Institute of Biochemistry and Medical Chemistry, Medical School, University of Pécs, 7624 Pécs, Hungary;
2 Department of Medical Biology and Genetics, Faculty of Medicine, Josip Juraj Strossmayer University of Osijek, 31000 Osijek, Croatia.
3 Institute of Physiology, Medical School, University of Pécs, 7624 Pécs, Hungary
4 Nano-Bio-Imaging Core Facility, University of Pécs, 7624 Pécs, Hungary
5 Hungarian Centre for Genomics and Bioinformatics, Szentágothai Research Centre, University of Pecs, 7624 Pecs, Hungary
6 Department of Theoretical Health Sciences and Nursing, Faculty of Health and Sport Sciences, Széchenyi István University, 9026 Győr
7 Department of Pharmacology, Faculty of Medicine, Josip Juraj Strossmayer University of Osijek, 31000 Osijek, Croatia
8 Clinical Institute of Laboratory Diagnostics, Osijek University Hospital, 31000 Osijek, Croatia

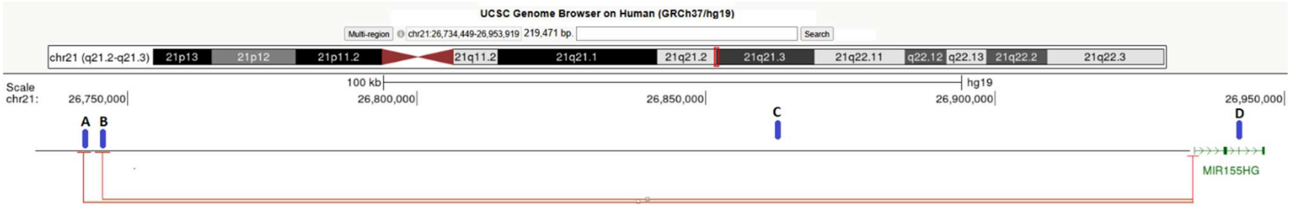

Figure S1. Genomic architecture and targeted ChIP-qPCR regions within the human *MIR155HG* locus. Schematic representation from the UCSC Genome Browser (GRCh37/hg19 assembly) mapping a ~220 kb window on chromosome 21 (q21.2–q21.3). Blue vertical bars designate the specific candidate regulatory regions selected for ChIP tracking, based on *in silico* JASPAR transcription factor binding analysis. Regions **A**, **B**, and **C** represent distinct distal upstream regulatory elements, while region **D** maps to a downstream intragenic site within the *MIR155HG* host gene body (green). Red interconnecting tracks at the bottom delineate GeneHacker interaction data, highlighting the physical long-range chromatin looping conformation between the distal upstream enhancers (**A** and **B**) and the core *MIR155HG* promoter.

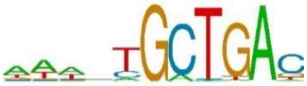

Figure S1B: Sequence of Consensus MAFB-bindig site

Supplementary Figure S1C: Consensus MAFB-Binding Sites and ChIP Primer Locations Potential MAFB binding sites are indicated in bold; ChIP primers are highlighted.

Region A - AAAGTGCTGAG

>hg19_dna range=chr21:26742747-26743211
ATGAGGTGGCCCAGAGCTCCCAGTGTTTCTGAGGCTGACTCAGAACTCTGAGTAGGACTGTGGTGAGCC
AACAGTCGCCATGGAATATGAACGAGACACATGGGTTTTGATGTGATGTGACCTCATGTGATGTGGTGT
AGCTGGAAGGGCCTCCTGGCACAGTGCTGGGCCCAGACTTGGGGGTCAGGACCTGTCAGATGAATTAGC
TTCTGAAAGAGTCAGCCATGCTGCCAGCAAACACTGTGGGTGGGCTTGGTATAGAGGATCGCCTGGTCA
GTGGTTCTCAAACTCGAGCATGTCAGAATGAACTGGTCTGCTGGTTAAAAATGCAGATTTCAGACCAGG
TGCAGTGGTTCACATCTGTACT**CTCAGCACTTT**GGGAGGCCGAGGCAGGTGGATCACTTGAGGCCAGGA
GTAAGAGACCAGCCTGGGCAGCATAGTGAGACCCTGTCTGAACAAAAAAAT
ChIP-A_F: TGACCAGGCGATCCTCTATAC
ChIP-A_R: CCCAAAGTGCTGAGAGTACAG

Region B
B_1) **AAACTGCTGA**A
B_2) CTAA**TGCTGAC**
>hg19_dna range=chr21:26745635-26745879
CAAAGGATCTTGTTTACCTTCTTTTTTTGTGGTCTAGAAAGGTCCTTATATAAGTGAACTACAGACTTC
TCTGTCCTCAGCTGAGACTCCTTTACTGAATGTTTGATTAAGCACCTTAAATACATTTTCCAGGTTCAA
G**AAACTGCTGAA**CACACCAGGTATAATGAGCTCAAGCATTGTGGGATCAAGATGAAACCTCAAACACAT
GGGAAATGCA**ACTAATGCTGAC**ATCTGTAGCAAAGGAA

ChIP-B_F: CAAGAAACTGCTGAACACACC
ChIP-B_R: TGCTACAGATGTCAGCATTAGT

Region C
C_1 AACTTGCTGAT
C-2 TCAATGCTGAC

>hg19_dna range=chr21:26862031-26862339
ctgatatttaaaaaCAAACTTGTTCTTCGGTTAATAGGGTTGAAAAAGGAAAAAAATTTGTTCCACATT
TATTAAACGAATGAATAAATTATGCTCTCTTTATTTCTATATC**ATCAGCAAGTT**CTTTGTGTTAATTTT
AAAATCTGGAATTGCTGT**GTCAGCATTGA**GACAAGTTGGAGAAGGAAAGCTGCTACTCCTGATTTGCTG
GCATTCCTCCTCCACGAGTCTGCACTCACTTCATGGTTTTGATATTCAAGTACAGCCTATTCATACACT
ATTCCCTCTGCAGAGAGGATCCTTTTTCTCCTT

ChIP-C_F: ATCATCAGCAAGTTCTTTGTGT
ChIP-C_R: TGCCAGCAAATCAGGAGTAG

Region D
D)AACAC**GCTGAC**

>hg19_dna range=chr21:26942096-26942454
ATAAACACATCTAAGGTGATGTGTCAACTTCAACCCCAACCCTTTTTCCCCAGATAACTTGTCTGCATT
TCAAGAACAACCTACCAGAGACCTTACCTGTCACCTTGGCTCTCCCACCCAATGGAGATGGCTCTAATG
GTAAGGAATTCTATCTTAAGTAAAAGGGAAGGGA**AACACGCTGAC**AGATGCAGTTTTTATAAAGGCAGG
TCATTGGGGCACCAAGTTGGCAGCCGCTTTCAACTTTTGATTCTACCTCAAAAGGTAACCAAGAAATCG

TTGTTATTCTAAAGATTATGACAAGTCCAAAGTTTCACAGAATCCTGATTTTTATACACTTGCATTAAT
CACTGCTTACTTCC

ChIP-D_F: GGAGATGGCTCTAATGGTAAGG
ChIP-D_R: CCCAATGACCTGCCTTTAT

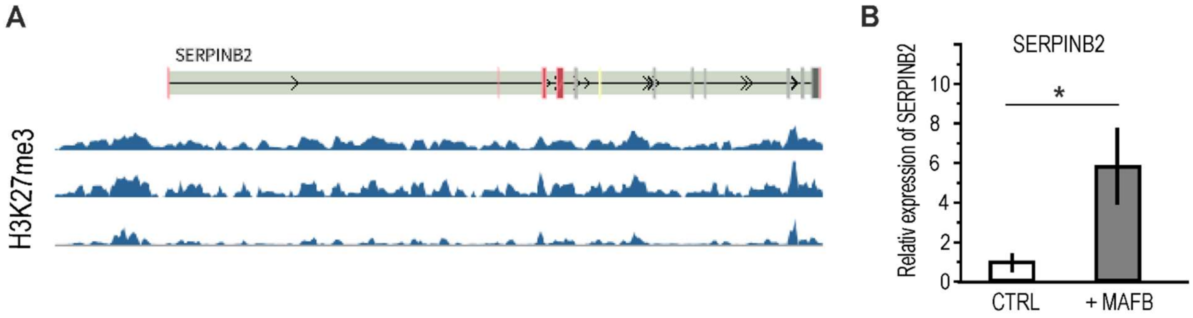

Figure S2. Baseline epigenetic silencing of *SERPINB2* by H3K27me3 and its *de novo* transcriptional induction following *MAFB* expression in HepG2 cells. **(A)** Public ENCODE ChIP-seq signal tracks showing enrichment of the repressive heterochromatin histone mark H3K27me3 across the human *SERPINB2* gene body and promoter region in baseline, unstimulated HepG2 hepatoma cells. Broad, continuous H3K27me3 signal peaks confirm that the *SERPINB2* locus is actively maintained in an epigenetically repressed heterochromatic state under homeostatic conditions. **(B)** RT-qPCR analysis of relative *SERPINB2* mRNA expression in HepG2 cells transfected with control vector (CTRL) or ectopic *MAFB* (+ MAFB). Ectopic *MAFB* expression overcomes baseline PRC2/H3K27me3-mediated repression, triggering a robust *de novo* induction of *SERPINB2* transcription (~6-fold vs. CTRL). Data are presented as mean SD (n = 3; *$p < 0.05$).
